# Two cases of placental trisomy 21 mosaicism causing false-negative NIPT results

**DOI:** 10.1186/s13039-023-00643-3

**Published:** 2023-07-14

**Authors:** Qinfei Zhao, Jing Chen, Ling Ren, Huijuan Zhang, Dedong Liu, Xuxiang Xi, Xiangsheng Wu, Chunyun Fang, Ping Ye, Shaoying Zeng, Tianyu Zhong

**Affiliations:** 1 Department of Laboratory Medicine, First Affiliated Hospital of Gannan Medical University, Ganzhou, Jiangxi China; 2 Department of Ultrasound, First Affiliated Hospital of Gannan Medical University, Ganzhou, Jiangxi China; 3 Department of Obstetrics and Gynecology, First Affiliated Hospital of Gannan Medical University, Ganzhou, Jiangxi China

**Keywords:** Non-invasive prenatal testing (NIPT), Down syndrome, False negative, Placental mosaicism, 21q;21q

## Abstract

**Background:**

Non-invasive prenatal testing (NIPT) using cell-free DNA has been widely used for prenatal screening to detect the common fetal aneuploidies (such as trisomy 21, 18, and 13). NIPT has been shown to be highly sensitive and specific in previous studies, but false positives (FPs) and false negatives (FNs) occur. Although the prevalence of FN NIPT results for Down syndrome is rare, the impact on families and society is significant.

**Case presentation:**

This article described two cases of foetuses that tested “negative” for trisomy 21 by NIPT technology using the semiconductor sequencing platform. However, the fetal karyotypes of amniotic fluid were 46,XY, + 21 der(21;21)(q10;q10) and 47,XY, + 21 karyotypes, respectively. Placental biopsies confirmed that, in the first case, the chromosome 21 placenta chimerism ratio ranged from 13 to 88% with the 46,XX, + 21,der(21;21)(q10;q10)[86]/46,XX[14] karyotype of placental chorionic cells (middle of fetal-side placental tissue). However, in the second case, of all the placental biopsies, percentage of total chimerism was less than 30%; and placental biopsies taken at the middle of maternal side and middle of fetal side, also had variable trisomy 2 mosaicism levels of 10% and 8%, respectively. Ultimately, the pregnancies were interrupted at 30 gestational age (GA) and 27GA, respectively.

**Conclusions:**

In this study, we present two cases of FN NIPT results that might have been caused by biological mechanisms, as opposed to poor quality, technical errors, or negligence. Clinical geneticists and their patients must understand that NIPT is a screening procedure.

## Background

Trisomy 21 (T21, also known as Down syndrome) is one of the most prevalent chromosomal abnormalities worldwide, occurring in approximately 1:700 live births [1]. Non-invasive prenatal testing (NIPT) has rapidly transformed the global prenatal screening landscape for common fetal chromosome aneuploidies because of its high sensitivity and specificity [2, 3]. NIPT evaluates the fetal cell-free DNA (cffDNA) fraction circulating in maternal blood, which can be detected at a gestational age (GA) as early as 9 weeks [4]. NIPT has been applied to screen high-risk patients for fetal aneuploidy as part of antenatal care and has increasingly been utilized in clinical practice.

Compared to other screening modalities, NIPT has unparalleled sensitivity and specificity for trisomy 21 [5, 6]. Over 99% of cases can be detected using NIPT, and the false-positive (FP) rate is less than 0.1% [7]. The cffDNA in maternal plasma originates from apoptotic cytotrophoblasts [8]; thus, in most pregnancies, the genetic components are identical between the placenta and fetal tissues. However, due to confined placental mosaicism, NIPT results may not always be representative of the true fetal karyotype, and both false-negative (FN) and FP results may occur [9–12]. Placental mosaicism [10], fetal chromosomal rearrangements, vanishing twin or co-twin demise [13], familial chromosomal abnormalities, and malignancy are common causes of FP NIPT results [14].

In contrast, among many clinical follow-up cases evaluated, FN NIPT results involving fetal aneuploidies have been rarely found [15, 16]. The presence of low cffDNA content and placental mosaicism has been associated with some FN findings, while others remain unexplained [17]. The effects of the aforementioned factors on FN NIPT results are unclear. Notably, there is a high possibility that FNs are clinically misdiagnosed, and the causes of FN NIPT results should be investigated. Clinical geneticists should recognize these FN results, and patients should be informed about discordant findings between NIPT and subsequent cytogenetic analyses.

This study reports two cases of fetal T21 associated with placental mosaicism that resulted in one FN NIPT result.

## Case presentation

### Case 1

**Fig. 1:**
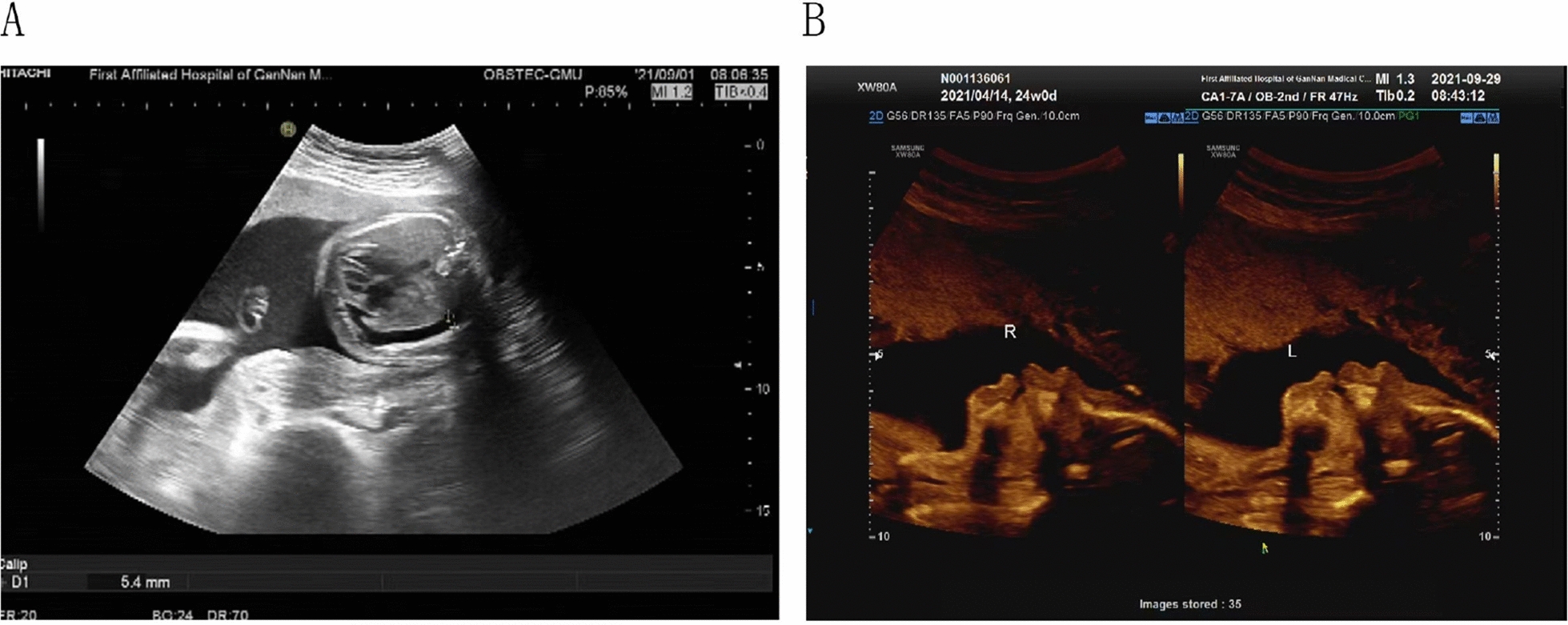
The ultrasound examination image. **A** Ultrasound examination result at 27 weeks. **B** Ultrasound examination result at 22 weeks

A 23-year-old healthy primagravida woman with a single fetus was referred to the First Affiliated Hospital of Gannan Medical University. A serological screening at 12 weeks combined with a nuchal translucency measurement (2.1 mm) suggested a critical risk for fetal T21 of 1 in 529. A NIPT examination at another hospital yielded a negative result at 15 weeks (fetal DNA fraction: 15.67%, chromosome 21 Z scores: − 0.201; Table 1). However, the patient was referred to our hospital at 27GA for routine ultrasonography, which showed that the fetus exhibited right-sided pleural effusion (Fig. 1A). Subsequently, the pregnant woman was referred to a hospital in the city of Guangzhou for further evaluation. The ultrasound scans showed bilateral pleural effusions and nasal dysplasia at 28GA. At 29 weeks, trisomy 21 of the fetus was identified via Quantitative Fluorescent Polymerase Chain Reaction (QF-PCR) and Chromosomal Microarray Analysis (CMA) by cordocentesis. The patient was transferred back to our hospital and underwent elective termination at 30GA after genetic counseling and communicating with family members. After gaining the consent from the patient, we retrieved the amniotic fluid, maternal peripheral blood, six placental biopsies (three from the fetal side and three from the maternal side), and umbilical cord tissue at termination and examined these samples in detail to understand the biological basis of the discrepancy.

**Table 1:** NIPT results for cases 1 and 2

Patient	Gestational Weeks	Unique reads/M	Fetal DNA Fraction (%)	NIPT Z-scores			NIPT result
				Chromosome13	Chromosome18	Chromosome21	
Case 1	15^a^	3.79	15.67	− 0.001	0.906	− 0.201	Low risk
	15^b^	8.54	14.89	− 0.432	− 0.01	0.951	Low risk
	29^c^	5.62	20.30	0.52	0.249	1.219	Low risk
	15^d^	9.86	20.10	− 1.22	0.93	0.566	Low risk
	29^e^	9.85	17.90	0.606	− 0.731	**3.414**	Critical risk of T21
Case 2	17	3.90	19.70	1.527	0.466	0.932	Low risk

Bold font indicates a high risk NIPT result

Z scores were calculated as previously described [18] with a normal range >  − 3 and < 3

a The Clinical laboratory data from the first examination

b The Clinical laboratory data from the reexamination

c The Clinical laboratory data before labor induction

d The third-party data from the first examination

e The third-party data before labor induction

**Fig. 2:**
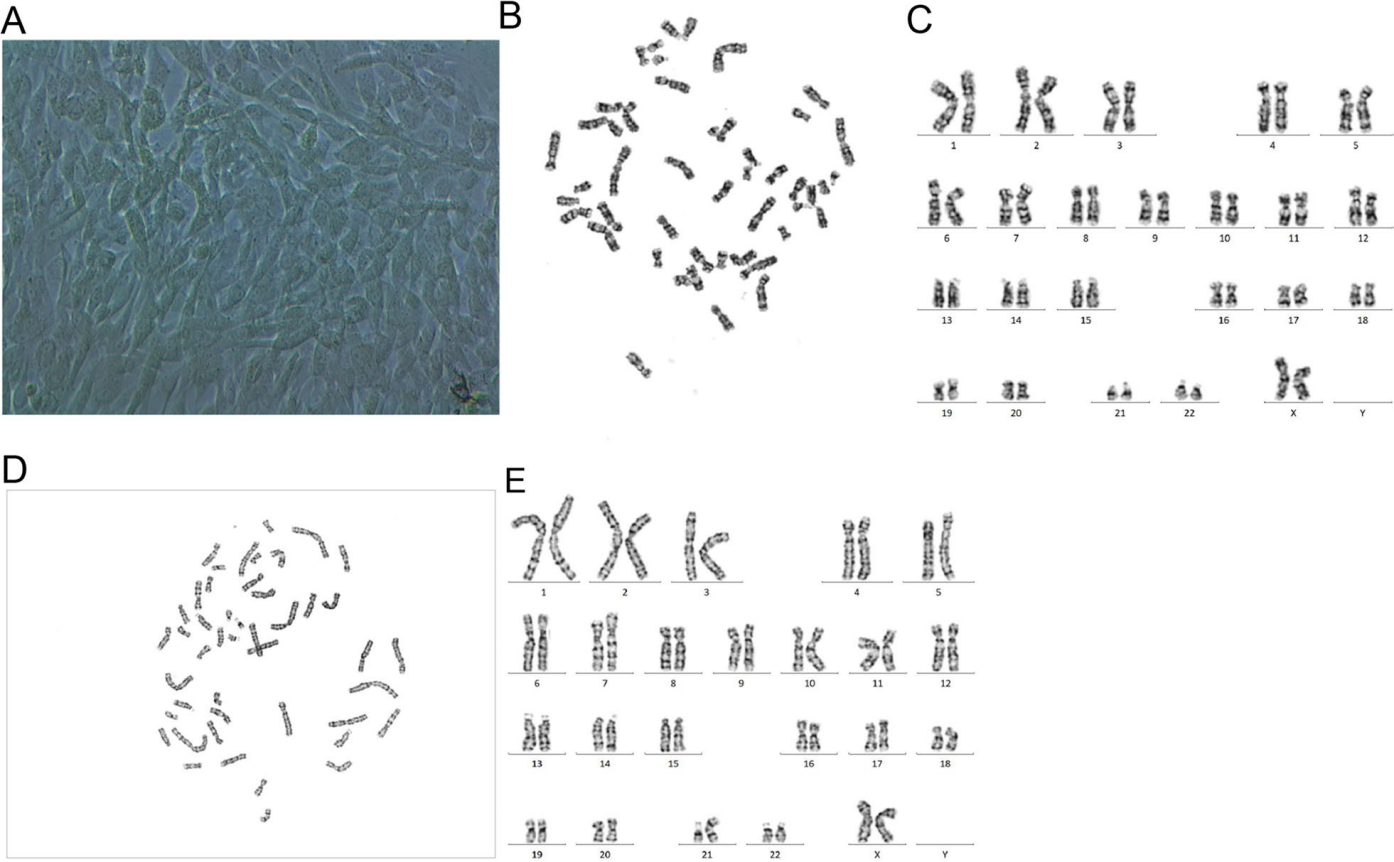
Morphology of placental chorionic cells and G-banded karyotypes. **A** Morphology of placental chorionic cells cultured on day 21 (× 40). **B**–**E** G-banded karyotypes of placental chorionic cells

**Fig. 3:**
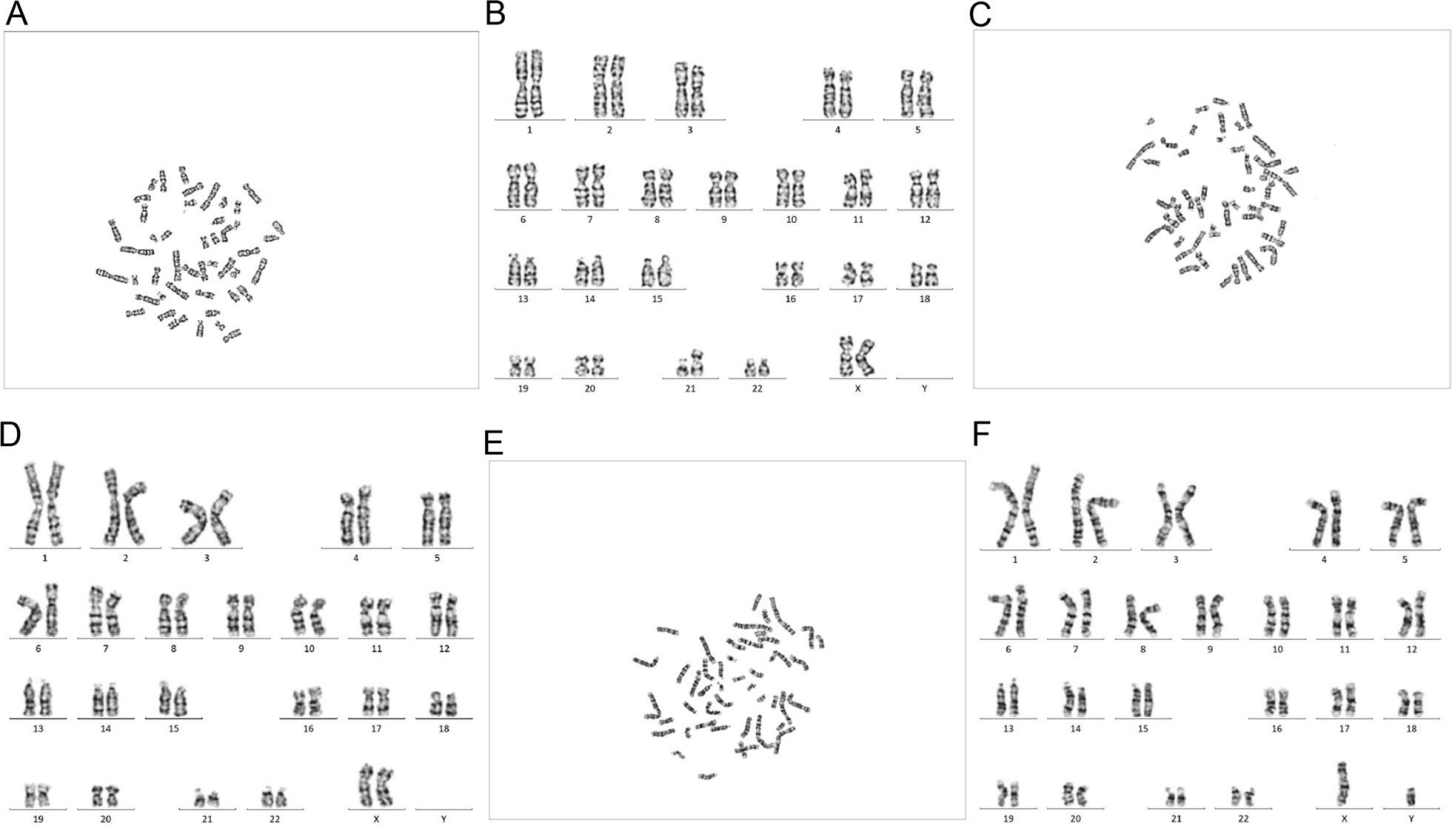
G-banded karyotypes of the fetus and his parent. **A** and **B** Fetus: 46,XX, + 21,der(21;21)(q10;q10); **C** and **D** Mother: 46,XX; **E** and **F** Father: 46,XY

As shown in Table 1, positive Z-scores were not detectable for chromosome 21 among five NIPT tests performed at different laboratories. Although the third-party data before labor induction was found to be greater than 3 (Table 1), the fetal concentration at that time was very high. Placental mosaicism may explain the false negative NIPT result, and no obvious problems were found in the clinical NIPT detection process. In addition, copy number variation using next-generation sequencing (CNV-seq) analysis results suggested that the degree of mosaicism of trisomy 21 varied greatly among different placental tissue sites; in particular, the proportion of mosaicism of trisomy 21 in maternal-side placental tissues was less than 30% (Table 2). Furthermore, the cytogenetics analysis of placental chorionic cells (middle of fetal-side placental tissue) demonstrated a mos 46,XX, + 21,der(21;21)(q10;q10)[86]/46,XX[14] karyotype, indicating that 86% of cells had trisomy 21 (Fig. 2), consistent with the CNV-seq analysis results of the placental tissue from the middle of the fetal side. However, the cytogenetic analysis of the amniotic fluid returned a karyotype of 46,XX, + 21,der(21;21)(q10;q10) without mosaicism, and both parents showed normal karyotypes (Fig. 3).

**Table 2:** CNV-seq analysis results

Patient	Experiment number	Subject	Sample type	CNV-seq results			Karyotype
				Chromosome 2 Z scores	Chromosome 21 Z scores	Speculated chimeric proportion of T21	
Case 1	1	Patient himself	Peripheral blood	− 1.287	− 1.545	46,XX	46,XX
	2	Center of maternal side	Placental tissue	− 0.224	26.864	47,XX, + 21[28%]	–
	3	Middle of maternal side	Placental tissue	0.275	16.952	47,XX, + 21[18%]	–
	4	Edge of maternal side	Placental tissue	− 1.128	20.968	47,XX, + 21[22%]	–
	5	Center of fetal side	Placental tissue	− 0.412	− 1.901	46,XX	–
	6	Middle of fetal side	Placental tissue	1.302	83.744	47,XX, + 21[88%]	46,XX, + 21,der(21;21)(q10;q10) [86]/46,XX[14]
	7	Edge of fetal side	Placental tissue	− 0.106	12.236	47,XX, + 21[13%]	–
	8	Root of umbilical cord	Umbilical cord tissue	0.355	88.798	47,XX, + 21[93%]	–
	9	Amniotic fluid	Amniotic fluid	–	–	–	46,XX, + 21,der(21;21)(q10;q10)
Case 2	1	Center of maternal side	Placental tissue	− 1.731	28.879	47,XY, + 21[30%]	–
	2	Middle of maternal side	Placental tissue	19.806	16.314	47,XY, + 21[17%]/47,XY, + 2[10%]	–
	3	Edge of maternal side	Placental tissue	− 0.576	20.569	47,XY, + 21[21%]	–
	4	Center of fetal side	Placental tissue	5.93	17.806	47,XY, + 21[19%]	–
	5	Middle of fetal side	Placental tissue	15.118	19.83	47,XY, + 21[21%]/47,XY, + 2[8%]	–
	6	Edge of fetal side	Placental tissue	3.818	17.52	47,XY, + 21[18%]	–
	7	Root of umbilical cord	Umbilical cord tissue	0.805	91.624	47,XY, + 21[96%]	–
	8	Amniotic fluid	Amniotic fluid	–	–	–	47,XY, + 21

### Case 2

A 35-year-old pregnant mother of two healthy children underwent a 17GA NIPT test that yielded a normal result (Table 1). An ultrasound examination at 22GA revealed fetal nasal bone dysplasia (Fig. 1B). After counseling, the couple underwent fetal testing by amniocentesis at 25 weeks, demonstrating a T21 fetal karyotype of 47,XY, + 21. In addition, the CMA results showed a pathogenic 15q11.2 microdeletion. The patient terminated her pregnancy at 27GA, and placental tissue was immediately collected for placental mosaicism analysis (Table 2). The CNV-seq analysis of the placental biopsies confirmed that the placental tissue had T21 mosaicism, with a chimeric ratio ranging from 17 to 30%, and the umbilical cord tissue had a chimeric ratio of 96% (Table 2). Notably, the placental tissue from the middle of the fetal side and the middle of the maternal side also showed T2 mosaicism, with chimeric ratios of 8% and 10%, respectively (Table 2).

## Discussion and conclusions

There is growing evidence that fetal DNA circulating in the maternal blood largely arises from placental trophoblastic cells, although fetal tissues also provide a small contribution [20]. Since cell-free DNA (cfDNA) was identified, NIPT has been widely promoted for prenatal screening for T21, T18, and T13 [21]. However, many factors may affect NIPT results, such as placental chimerism, maternal obesity, and maternal cancer [22]. In general, FN results are likely caused by two factors. First, if the proportion of cffDNA does not meet a certain value, the positive signal may be indistinguishable from the background signal. Second, due to placental chimerism, the plasma cffDNA can be derived from an area of the placenta with either no chimerism or a low proportion of chimerism. Due to advances in cfDNA enrichment techniques, NIPT can achieve lower detection limits than previous approaches. Confined placental mosaicism is the main reason that leads to FP or FN results with NIPT [10]. Placental mosaicism refers to a karyotype difference between placentally and fetally-derived tissues [23]. In this study, we provide information about two rare cases of FN NIPT results with partial T21 caused by placental mosaicism. This situation should be known to clinical professionals, and patients should be informed that discordant NIPT results may occur.

In the first case of placental mosaicism, multiple plasma experiments and CNV-seq analyses of distinct areas of placental tissue revealed that the NIPT negative results are likely attributed to the low placental mosaicism. However, amniotic fluid cytogenetic analysis revealed 46,XX, + 21,der(21;21)(q10;q10) without mosaicism, and both parents had normal karyotypes. Accordingly, this 21q;21q rearrangement was a de novo fetal chromosomal 21q rearrangement. According to some related research reports, most 21q;21q rearrangements are isochromosomes [24], and Down syndrome resulting from a de novo isochromosome 21q is more likely to lead to a FN NIPT result than standard karyotypes (47,XN, + 21) [25, 26]. Interestingly, the karyotype of placental chorionic cells (derived from the placental tissue from the mid-fetal side) was 46,XX, + 21,der(21;21)(q10;q10)[86]/46,XX[14]. To the best of our knowledge, this study investigates placental mosaicism from a cytogenetic perspective for the first time [25]. These results indicate that placental mosaicism caused by 21q;21q rearrangements is almost certainly a biological cause of FNs.

Regarding placental mosaicism in the second case, the CNV-seq analysis revealed a low T21 mosaicism percentage in all the different regions of placental tissue tested. Unexpectedly, placental biopsies taken from the middle of the maternal side and the middle of the fetal side also had variable T2 mosaicism levels of 10% and 8%, respectively. Altogether, the percentage of total chimerism was less than 30% in all the placental biopsies. The above results indicated that the NIPT negative results are also likely attributed to the low placental mosaicism.

In order to examine the correlation between the mosaic proportions of placental tissue and the Z-score for T21 of the NIPT, a search was conducted for published cases of false negative NIPT results due to T21. Regrettably, the majority of FN NIPT cases did not identify the placental biopsy tissues. Ultimately, a total of five FN NIPT cases were collected (Table 3). The current study's results indicate that the Z-score for T21 of the NIPT does not consistently reflect the T21 level present in placenta (Table 3). These findings have implications for both clinicians and patients, as they highlight the complexity of cfDNA screening biology.

**Table 3:** Published cases of false negative NIPT results due to T21

Case number	cfDNA screening technology	Indication for NIPT	Pregnant woman age (yrs)	Blood drawn at GA (wk + d)	Fetal DNA fraction	Z-score for T21	Karyotype	Explanation for false negative NIPT result	Study [reference]
1	MPSS	1/370 risk for T21 by serum screening	32	18 + 0	15.60%	2.04	46,XX,der(21;21)(q10;q10), + 21	Placental biopsies had 17–53% with T21 mosaicism	Wang et al. (2013) [9]
2	MPSS	1/529 risk for T21 by serum screening	23	15	15.67%	− 0.201	46,XY,der(21;21)(q10;q10), + 21	Placental biopsies had 13–88% with T21 mosaicism	This study
3	MPSS	Ultrasound markers	35	17	19.70%	0.932	47,XY, + 21	Placental biopsies had 17–30% with T21 mosaicism	This study
4	MPSS	Ultrasound markers	35	18	19.72	1.33	47,XY, + 21	Placental biopsies had 2.6–76% with T21 mosaicism	Wang et al. (2013) [9]
5	tMPS	a history of multiple adverse pregnancy outcomes	37	16 + 5	7.52%	2.503	47,XX, + 21[22]/46,XX[4]	Placental biopsies had about 42.9%% with T21 mosaicism	Kang et al. (2022) [19]

*MPSS* Massive parallel shotgun sequencing; *tMPS* targeted massive parallel sequencing

The cases discussed here emphasize the importance of and the necessity for the complementary ultrasonographic control when NIPT results are negative. Therefore, clinicians and patients must understand that NIPT is a screening test. Individuals with negative NIPT results should be provided regular ultrasound monitoring to prevent misdiagnoses and should undergo further prenatal diagnostics, if necessary.

## Data Availability

All key data generated during this study are included in this published article.
